# Supplementary material for: Phytochemicals Prime RIG-I Signaling and Th1-Leaning Responses in Human Monocyte-Derived Dendritic Cells
Source: Nutrients. 2025 Nov 12;17(22):3539. doi: 10.3390/nu17223539 (PMC12655279; doi:10.3390/nu17223539)
Supplement: Supplementary file 1 [file nutrients-17-03539-s001.zip › nutrients-3933843-supplementary.pdf]

Supplementary Materials:

Table S1. qPCR primers sequences

Gene	Primer sequence 5′-3′
GAPDH	(F)5′-ATGGGGAAGGTGAAGGTCG-3′<br>(R)5′-GGGGTCATTGATGGCAACAAT-3′
RIG-I (DDX58)	(F)5′- CACCTCAGTTGCTGATGAAGGC -3′<br>(R)5′- GTCAGAAGGAAGCACTTGCTACC -3′
TLR9	(F)5′- CAACAACCTCACTGTGGTGC-3′<br>(R) 5′- TCCTTCAACACCAGGCCTTC-3′
IRF-7	(F)5′-CCACGCTATACCATCTACCTGG- 3′<br>(R)5′-GCTGCTATCCAGGGAAGACACA-3′
IFN-β	(F)5′-ATGACCAACAAGTGTCTCCTCC-3′<br>(R)5′-GCTCATGGAAAGAGCTGTAGTG-3′

Figure S1. Purity of $CD2^+$/ $CD56^-$ T cells

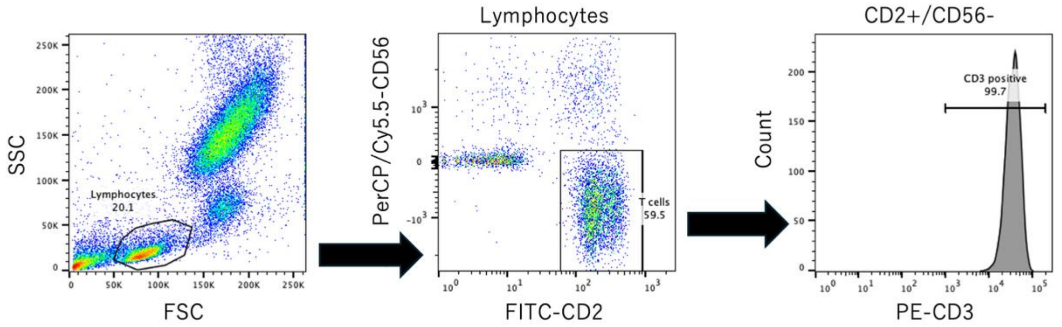

Figure S1. CD3 positivity in T cell sorting. Whole blood was fluorescently stained with CD2 (FITC), CD3 (PE), and CD56 (PerCP-Cy5.5), and sorted using FACSMelody. CD3 was 99.7% positive in $CD2^+$ T cells. The purity of T cells was assessed using the following fluorescently labelled anti-human antibodies: CD2-FITC (TS1/8) (BioLegend), CD56-PerCP/Cyanine5.5 (HCD56) (BioLegend), and CD3-PE (OKT3) (BioLegend).

Figure S2. pDC qPCR Relative Quantity

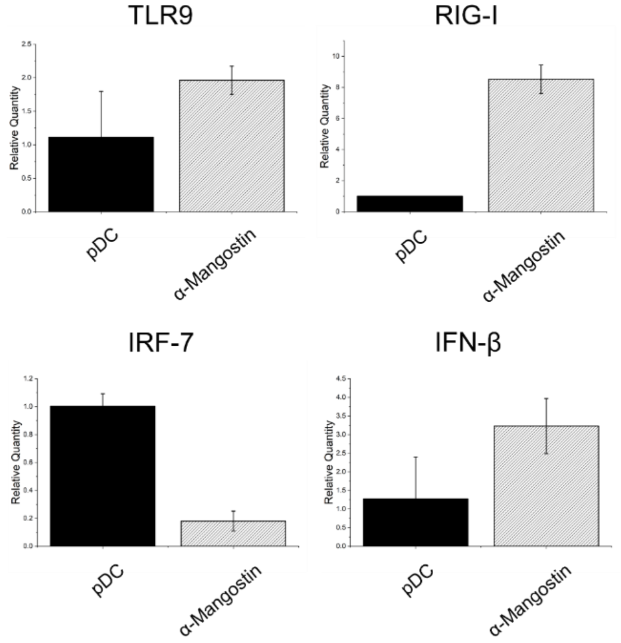

Figure S2. Phytochemical-induced gene expression levels in pDCs. pDCs were isolated from PBMCs via FACS sorting. Phytochemicals were added to pDCs at 40 μM each and cultured for 2 h. Gene expression levels of *TLR9*, *RIG-I*, *IRF-7*, and *IFN-β* were measured by qPCR.
